# Developmental delay in the Amazon: The social determinants and prevalence among rural communities in Peru

**DOI:** 10.1371/journal.pone.0186263

**Published:** 2017-10-12

**Authors:** Christopher Westgard, Yossef Alnasser

**Affiliations:** 1 Department of Innovative Research, Asociación Red Innova, Lima, Perú; 2 British Columbia Children’s Hospital, University of British Columbia, Vancouver, Canada; TNO, NETHERLANDS

## Abstract

The consequences of poor child development are becoming increasingly recognized. Programs are being put in place around the world to improve child development by providing healthy and stimulating environments for children. However, these programs often have limited reach and little is known about the prevalence of developmental delay in under-developed communities. The current study set-out to better understand the prevalence of developmental delay in rural communities in the Amazon region of Peru. Also, it explores social determinants that are associated with any delay. Cross-sectional study by evaluating developmental delay in children under 4 years utilizing Ages and Stages Questionnaire (ASQ-3). Additionally, conducting a social determinants questionnaire answered by caretakers to identify social drivers for developmental delay. The data was analyzed with multi-variant analysis to measure association. The prevalence of developmental delay in the Amazonian communities was 26.7% (19.3% in communication, 11.4% in gross motor skills, 8% in both) (N = 596). The multivariate logistic regression analysis revealed significant associations between developmental delay and; level of education (OR 0.64, p = 0.009), age of mother during child’s birth (OR 0.96, p = 0.002), visits by community health agents (OR 0.73, p = 0.013), and river as primary water source (OR 2.39, p = 0.001). The social determinants questionnaire revealed that 39% of the mothers had their first child before the age of 17, nearly half stopped going to school before the age of 12 (52%), 29% gave birth at home, 13% breast fed for less than 7 months, and 50% of the children had diarrhea in the last month. There is still a great need to improve the conditions for child development in the Amazon region of Peru. One-fourth of the children suffer from developmental delay, which will likely impede their potentials for life unless something is done. The impact of education, age of mother at birth of the child, community health agents, and access to clean drinking water were important findings. Improvements can be made in these areas to create a large, cost-effective impact on the well-being of the communities.

## Introduction

“Child development refers to the ordered emergence of interdependent skills of sensori-motor, cognitive language, and social-emotional functioning” [1]. The importance of child development is becoming more recognized and prioritized in developing countries around the world. Many developing countries have promoted infant stimulation programs, preschool programs, and effective parenting programs [1–5]. In Peru, a national program was implemented to send representatives of the Ministry of Social Inclusion to visit caretakers in distant communities to provide health messaging and child stimulation (Cuna Mas) [6].

It is known that there is a need to prioritize good child development because poor development has several long-term consequences, such as poor school performance, low wages, and high fertility rates [7]. However, there are limited local, regional, or even national statistics that display the burden of developmental delay. The burden of poor child development can be estimated based on exposure to risk factors, such as poverty, stunting, inadequate cognitive stimulation, iodine deficiency, iron deficiency anemia, intrauterine growth restriction, and others [8]. One study analyzed the global statistics of stunting and extreme poverty to estimate the worldwide burden of poor child development to be over 200 million children [7]. Unfortunately, the estimation cannot take into account all local and cultural drivers of child development, which likely leads to underestimation of the burden.

The current study takes place in Peru, where there are limited national statistics regarding child developmental delay. A study was done by Kyerematen, et al., [2014] in a low-income urban zone of Lima, Peru that found 38.7% of children examined (N = 129) are suspect to developmental delay in at least one of the developmental categories [9]. Another study in Peru, [10] found that the scores from the Ages and States Questionnaire (ASQ) for children in the fifth wealth quintile were significantly lower than those in the first wealth quintile (.27 of standardized score) [10].

As far as we know, this is the first study to assess prevalence of developmental delay in the Amazonian Basin of Peru. Additionally, it is the first to explore social determinants of developmental delay in those communities. In this study, the research team set-out to measure the prevalence of developmental delay, measure various social determinants, and analyze the data to identify associations between social determinants and developmental delay.

## Materials and methods

### Location and population

The study took place in the Amazonian departments of Loreto and Ucayali, Peru. The region is home to a large diversity of indigenous groups. In the seven districts that were included in the study, seven distinct Amazonian ethnic groups are represented. These include; Ashaninka, Cocama, Shipbo-Conibo, and Piro in Ucayali, and Yagua, Orejon, and Quichua in Loreto [11].

For the study sites, 15 communities were included, 8 in Ucayali and 7 in Loreto. The communities were chosen by the mayors of each district, and had to satisfy the inclusion criteria of previously implementing the program, Community Center for Outreach and Surveillance (Centro de Promocion y Vigilancia Comunal) [12,13]. The mayors of each district were approached during a regional conference and asked if they wanted to participate in the study, the districts that showed interest where included. The list of communities included in the study are shown in Table 1, including number of children age 1 to 3 and number of participants included in our study. The map in S1 Annex displays the location of each district included in the study.

**Table 1:** Communities included in the study, population of children, and sample size obtained from each community.

Department	Province	District	Community	Population of Children age 1–3	Sample Size
Ucayali	Coronel Portillo	Masisea	Santa Rosa de Masisea	36	30
		Iparia	Iparia	234	81
	Padre Abad	Irazola	San Alejandra	335	99
		Campoverde	Nueva Tunuya	7	12
			La Victoria	60	45
	Atalaya	Sepahua	Sepahua	287	66
			Bufeo Pozo	78	25
			Puija	38	11
Loreto	Maynas	Las Amazonas	Fransico de OrenallA	106	19
			Oran	90	43
			Yanashi	54	41
		Indiana	Indiana	155	36
			La Libertad Vainilla	19	18
		Mazan	Mazan	215	74
			La Libertad	16	11
			**TOTAL**	**1496**	**611**

The study participants were children between ages of 8 months to 38 months, and their caretakers. The age group was determined to assess early child development which can be measured by using, Ages and Stages Questionnaire, with good accuracy [14]. Also, to catch children while they are still involved in early child development activities (breast feeding, consuming micronutrients, growth monitoring check-ups). The date of birth of each child was verified with caretaker then confirmed by child’s identification card. If an identification card was not available, the surveyor wrote the date of birth as indicated by the caretaker.

To determine the minimum sample size needed, authors considered the total number of children within the age range in the selected communities and utilized the sample size calculator of EpiInfo 7.2 StatCalc. The calculation included the total population of 1496, an expected frequency of 25%, Acceptable Margin of Error at 5%, and a design effect of 1. To reach a 99.9% confidence level, the minimal sample size had to be 527 children. All children and their caretakers in each community were sought to be included in the study. The research team visited all houses in each community. Many families were not home because they were traveling. We found that the demographic information regarding the population of the community (displayed in Table 1 and provided by the mayors of each district) was often incorrect or included the population of several surrounding communities.

### Data collection

The data collection was conducted by a team of psychologists from the region and the primary investigator at the beginning of 2017. The team of researchers went door to door asking if a child at the age of 8 months to 38 months and their caretaker was home. Caretakers who consented to participate in the study were included.

The interviewer first conducted a social determinants questionnaire with the caretaker that consisted of 32 questions. The questionnaire included questions regarding demographics, home conditions, brief medical history, health related behavior for caretaker and child, and use of health services. The questions were chosen based on their expected relationship with child development and malnutrition [8,15,16].

The wealth index was created to estimate the wealth of the family based on the presence of desired amenities. The amenities were chosen based on their availability in all communities, sufficient variability in the households, (at least 10% and at most 90% of households have them), and display a status of wealth in the communities. Ownership of a television, cellular phone, and radio set as indicators of wealth. The wealth index was created by receiving one point for each appliance.

The study utilized the Ages and Stages Questionaire-3 (ASQ) to screen for developmental delay in the children. The ASQ has been validated as an effective assessment tool for developmental delay in a wide-range of contexts and is significantly less time consuming and more inexpensive to administer [17]. The ASQ is a feasible evaluation tool for large scale deployments for prevalence studies and program [10,14,18,19]. The ASQ has been validated for test-retest in Spanish, including in the context of the Amazon of Peru, and found reliability scores of .80-.85 (differing by section)[18]. The Spanish version was purchased and used for the current study. An initial field trail was conducted in the Amazon region to assess the relevance of language and examples presented in the ASQ. The examples and language were culturally relevant with appropriate explanation from the evaluator [17,20]. Psychologist from the region (Pucallpa, Ucayali) assisted in the field validation.

The ASQ was shortened to decrease the time and materials needed to complete the exam. The surveyors were tasked to observe the behavior being assessed in the child whenever possible (as opposed to only asking the caretaker) to improve the accuracy of the assessment and minimize bias from the parent’s recall. The research team wanted to deploy an exam that could be used at scale for large demographic surveys and evaluation programs. To do so, the authors shortened the questionnaire to include only two sections, communication and gross motor. Furthermore, communication and gross motor sections of the ASQ-3 have the highest test-retest reliability (communication = .92, gross motor = .9, problem solving = .8, fine motor = .37, personal-social = .73)[14]. Also, communication and gross motor sections have the highest correlation with Bayley-III (the gold standard) [14].

The interviewer performed the ASQ with the caretaker and the child. For each question, the interviewer asked the caretaker if the child could perform the task. If the caretaker answered yes, her child was asked to demonstrate the given task. If the child could perform the task they received 10 points for that question, if they could perform the task part of the time they received 5 points, if they could not perform the task they received 0 points. In the case that a child was not willing to participate in the activity, the interviewer accepted the caretaker’s response directly. At the end of each interview, the results of the ASQ were reported to the caretaker with recommendations of activities to encourage child development. All interviewers received a formal training course in how to conduct the interviews prior to deployment to avoid any bias and observed examples in the field to standardized responses.

### Data analysis

The scores from the ASQ evaluation of each child were compared to the cutoff score provided by ASQ that determines if the child is experiencing developmental delay and requires further assessment. If the child was delayed in one of the two categories (communication or gross motor), they received a score of 1, and if there was no delay detected, they received a score of 0.

The results of the social determinants questionnaire were organized in a spreadsheet using Excel, and analyzed by Epi Info 7. To find interrelationship between social factors and developmental delay two multivariate analysis were conducted, a full model analysis and a restricted model analysis were employed. The full model analysis was run with all predictor variables that could have an impact on nutrition and child development [1,8,15,21]. The exclusion criteria included omitting the variables that did not provide enough variance (illness of the mother during pregnancy, illnesses of child, hours of electricity) or did not have reliable data (months of exclusive breastfeeding, birth weight, deworming, vaccinations). The unreliable data was a result of lack of knowledge by the caretaker or misunderstanding the question. All the remaining variables were included in the full model to get a complete picture of their associations. However, the full model omits a large number of observations (*N* = 236). The restricted model analysis omits two predictor variables to increase the sample size of the analysis to 589. For the restricted analysis, we omitted the variable that measured how long the mother breast fed the child, and how long they gave their child micronutrient supplement. All children that still breastfeed or receive micronutrient supplement could not be included in the analysis because we do not know the age in which they stopped being breastfed or given supplements. The two variables were not statistically significant in the full model. Based on a likelihood ratio test, the full model is no better at predicting the outcome (1.71, p = 0.425). Additionally, the full model omits a large portion of the youngest population of the sample, which is of great importance. Therefore, the results from the restricted analysis are presented as primary findings in this study.

### Ethics

The study was approved by the Research and Institutional Ethics Committee of the National Hospital San Bartolome (Hospital Nacional Docente Madre Niño “San Barolome”). The consent form was written and presented in Spanish. Families sometimes translated the consent form to older members of the household that did not speak Spanish. The head of the family of each participant signed the consent form. The study findings were immediately communicated back to all participating caretakers.

## Results

### Participation rate

The ASQ was conducted on 593 children between the ages 8–39 months. The social determinants questionnaire was conducted on 605 caretakers between the ages 13–46 years old. A total of 12 children did not receive the ASQ from the study population (98% response rate) because they were uncomfortable with the presence of the interviewer or the interview was cut short (data from caretakers was still included). Of the total population of communities surveyed, 40.8% of the children were included. The highest participation rate was in the community of Masisea (83%) and the lowest was in the community of Sepahua (25.3%).

### Development delay prevalence

**Fig 1:**
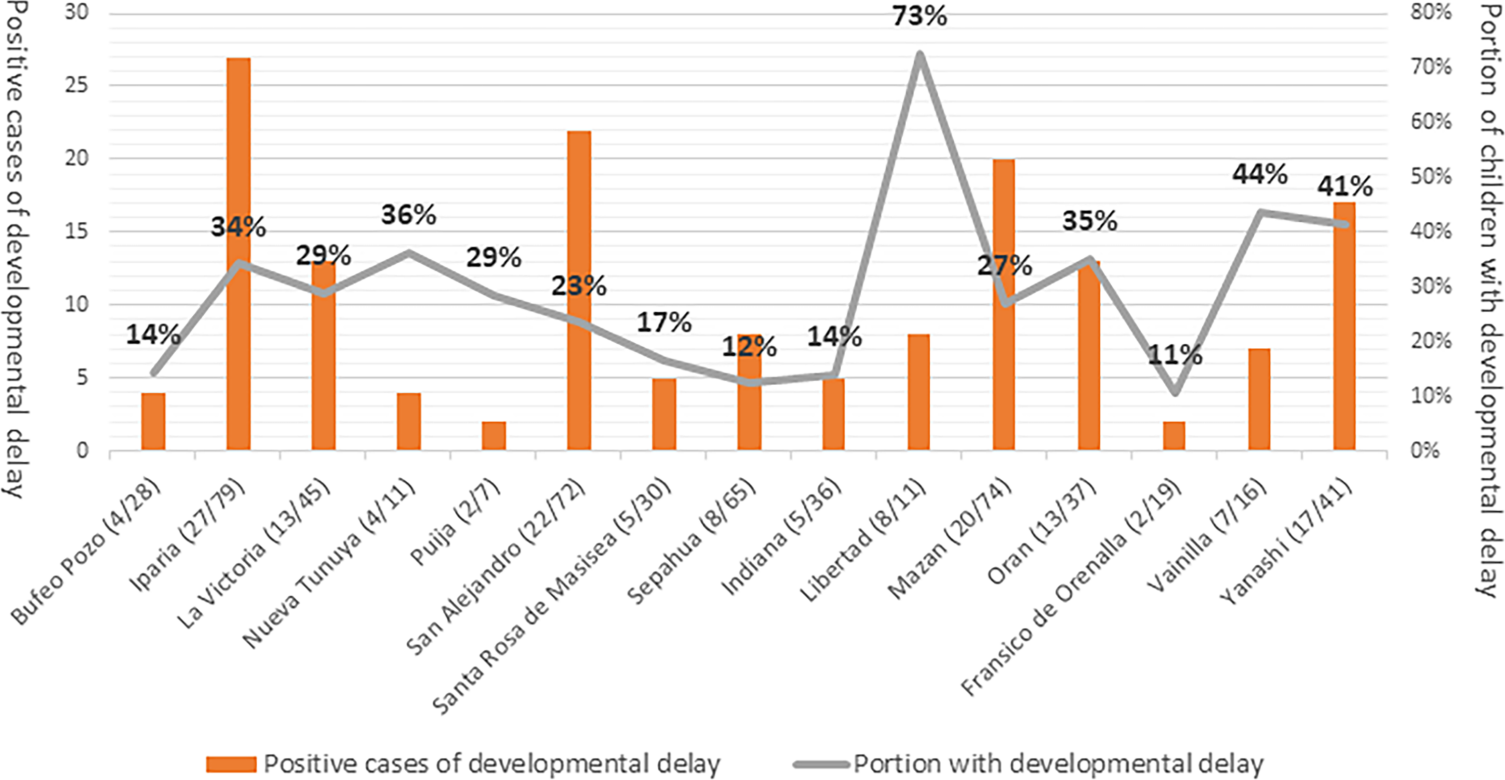
Prevalence of developmental delay in each participating community among Amazonian communities of Peru.

The prevalence of developmental delay as observed by the ASQ in the communities was 26.7% (159/593). Of the children examined, 19.3% had developmental delays in communication, 11.4% had developmental delays in gross motor skills, while 8% had developmental delays in both. **(Fig 1)**

### Social determinants

The descriptive statistics of the social determinants are presented in Table 2. We found that 39% of the mothers had their first child before the age of 17, 52% stopped going to school before the age of 12, 29% gave birth at home, 13% breastfed for less than 7 months, and 50% of the children had diarrhea in the last month. We also found that 80% took maternal vitamins during their last pregnancy, 76% gave their child micronutrient powder, 83% take their child to growth monitoring check-ups, and 51% spoke to a community health agent in the last month.

**Table 2:** Descriptive statistics of study population, by department.

Characteristics	Loreto (N = 236)		Ucayali (N = 360)		Total (N = 596)	
	Mean (SD)	Percent	Mean (SD)	Percent	Mean (SD)	Percent
Child has developmental delay	-	30.5% (N = 72)	-	24.2% (N = 87)	-	26.7% (N = 159)
Child has no developmental delay	-	69.5% (N = 164)	-	75.8% (N = 273)	-	73.3% (N = 437)
Child is male	-	50.8%	-	53.9%	-	53.0%
Child's age (mo)	22.9 (8.8)	-	24.5 (9.23)	-	23.9 (9.1)	-
Number of Children in family	3.1 (1.8)	-	2.8 (1.9)	-	3.0 (2.2)	-
Mother's age at child's birth	25.8 (7.6)	-	24.9 (7.5)	-	25.2 (7.5)	-
Mother's age at first birth before 17 years old	-	39.6%		38.9%	-	39.2%
Mother completed her education before 12 years old	-	62.2%	-	46.5%	-	52.4%
Gave birth at home	-	29.9%	-	29.5%	-	29.7%
Mother took maternal vitamins during pregnancy	-	91.7%	-	72.2%	-	80.0%
The mother had Malaria during pregnancy	-	4.6%	-	0.0%	-	1.8%
Wealth Index ^a^	-	17.4%	-	32.8%	-	26.7%
Fed with breast milk for 6 months or less	-	6.9%	-	17.6%	-	13.2%
Goes to child growth monitoring checkup	-	93.8%	-	76.3%	-	83.4%
Child was given micronutrients	-	90.1%	-	66.4%	-	75.9%
Duration of micronutrients	13.8 (9.8)	-	7.7 (9.1)	-	9.5 (9.7)	N/A
Child has taken a deworming pill ^b^	-	43.5%	-	36.9%	-	40.0%
Child has received their vaccines	-	86.3%	-	82.8%	-	84.3%
Child had diarrhea in the last month	-	50.8%	-	47.9%	-	50.9%
Spoke with a Community Health Agent in the last month	-	63.6%	-	43.0%	-	51.3%
Visited the Community Center for Outreach and Surveillance	-	48.0%	-	16.0%	-	28.7%
Home has Sanitary Toilet ^c^	-	19.8%	-	23.3%	-	22.1%
Water source is the River	-	46.7%	-	0.0%	-	18.7%

^a^ Has television, radio, and cell phone

^b^ Only Children over 18 months old included

^c^ Bathroom with running water, latrine with septic tank, or decomposing toilet system

For the logistical regression analysis, the results are displayed in Table 3. The full model analysis (N = 329) included 13 variables, of which 4 were found to be significantly associated with developmental delay: the level of education of the mother (OR = 0.56, p = 0.012), age of mother at birth of child (OR = 0.95, p = 0.007), number of days child had diarrhea in the last month (OR = 0.006), and river as primary source of water (OR = 2.39, p = 0.029). The restricted model analysis (N = 589) included 11 variables, of which 4 were found to be significant: the level of education of the mother (OR = 0.65, p = 0.009), age of mother at birth of child (OR = 0.96, p = 0.002), number of home visits received by community health agents (OR = 0.73, p = 0.013), and river as primary source of water (OR = 2.79, p<0.001).

**Table 3:** Logit regression: Association and odds ratio between developmental delay and each social determinant.

Characteristics	Full Analysis (N = 329)			Restricted Analysis (N = 589)		
	OR	P	95% CI	OR	P	95% CI
Sex (male)	1.46	0.181	0.83 2.54	1.46	0.181	0.83 2.54
Level of Education	0.56	0.012*	0.44, 2.49	0.65	0.009*	0.48, 0.89
Age of mother at birth of child	0.95	0.007*	0.91, 0.98	0.96	0.002*	0.93, 0.99
Gave birth in home	0.73	0.37	0.38, 1.41	0.88	0.588	0.56, 1.39
Took maternal vitamin, months	0.99	0.91	0.89, 1.11	0.94	0.078	0.87, 1.01
Goes to growth monitoring checkups	0.49	0.053	0.25, 1.01	0.63	0.073	0.37, 1.05
Community health agent visits	0.83	0.34	0.56, 1.22	0.73	0.013*	0.56, 0.94
Child with diarrhea, days	1.12	0.006*	1.03, 1.21	1.04	0.154	0.98, 1.10
Breast fed child, months	0.98	0.357	0.93, 1.03	-	-	-
Gave child micronutrient powder, months	1.02	0.338	0.99, 1.05	-	-	-
Wealth Index						
-0 devices	1	-	-	1	-	-
-1 device	1.61	0.252	0.71, 3.67	1.77	0.070	0.96, 3.29
-2 devices	1.11	0.811	0.46, 2.68	1.58	0.151	0.85, 2.95
-3 devices	0.53	0.19	0.20, 1.39	1.09	0.810	0.55, 2.15
Bathroom						
-In House Plumbing	1	-	-	1	-	-
-Latrine	1.05	0.917	0.44, 2.49	0.76	0.347	0.43, 1.35
-Open Defecation	1.45	0.506	0.49, 4.27	1.03	0.942	0.51, 2.08
Water Source						
-Home Tap System	1	-	-	1	-	-
-Well Water	2.17	0.105	0.85, 5.35	1.36	0.376	0.69, 2.69
-River Water	2.39	0.029*	1.09, 5.25	2.79	0.000*	1.63, 4.79

*p<0.05

A Correlation Matrix of Coefficients of Regress Model was conducted to assess correlation between the independent variables and ensure there was no multicollinearity. The highest correlation between independent variables was between *Community Health Agent Visits* and *Visits Community Center for Outreach and Surveillance* (r = 0.38). Therefore, *Visits Community Center for Outreach and Surveillance* was dropped from the analysis. The next highest correlations between independent variables was low, including: *Location of birth* and *Type of bathroom* (r = .29), and *Took maternal vitamins* and *Water source* (r = .195), and so they remained in the analysis. With the exclusion of *Visits Community Center for Outreach and Surveillance t*he interdependence between independent variables was found to be minimal.

## Discussion

Cultivating good childhood development of cognitive and socioemotional skills has a large and long-term impact on health, wealth, and well-being of the person and society [1,7]. Poor early childhood development has been estimated to create $177 billion of economic loses in low- and middle-income countries [22]. Peru has high prevalence of several risk factors that are associated with poor child development. The national average of chronic malnutrition is 14.6% and anemia is 35.6% for children under the age of five [23]. Unfortunately, these risk factors are more prevalent among indigenous communities in the Amazonian region [24–26]. In the Amazonian department of Ucayali, chronic malnutrition is at 26.1% and anemia is at 46% [23]. Two independent studies found chronic malnutrition rates among children in indigenous communities to be 50% and 56.2%, and anemia rates to be 51% and 51.3% [24,25].

Through the application of the ASQ, we found that more than one-fourth of the children [26.7%] in the studied communities are experiencing development delay. The probability of a child having developmental delay was significantly associated with several factors. If the family uses the river as their primary source of drinking water the child is more likely to experience developmental delay (OR 2.81). The association is important because 19% of the families interviewed still drink directly from the river (47% of families in Loreto). Mothers’ education showed protective effect against development delay among children. For every additional year of education for mothers, the probability of the child experiencing developmental delay decreased [OR 0.65). Our findings reflected other studies that found that mothers intellectual ability (measured by years of education or test scores) can have a strong impact on child development and academic performance [27,28]. However, we found that 52% of the caretakers in the communities stopped going to school before the age of 12. We also found comparable outcomes in relation to the impact of adolescent pregnancy. In our study, young mothers were more likely to raise a child with developmental delay (OR 0.96). Adolescent pregnancy is a large problem in the communities, where 39% have their first child before the age of 17. The outcome has been reflected in other studies in developed and developing countries. A 16-country study found that girl child marriage is a significant risk factor for early childhood developmental delay and stunting [29]. A mitigated factor for developmental delay was found in home visits by a community health agent. The families that received home visits were less likely to have a child with developmental delay (OR 0.89). However, only 51% of the families in the communities currently receive visits by a community health agent. The average is probably much lower in the general population of the region, as we surveyed communities that have a Community Center for Outreach and Surveillance and so are more likely to have community health agents [12,13].

The prevalence of developmental delay in the Amazon region obtained in the current study is more elevated then the prevalence obtained in the prior study by Kyerematen, et al., [2014] in urban slum communities (19.1% in communication and 11.1% in gross motor vs. 15.5% in communication and 3.8% in gross motor, respectively) [9]. This is consistent with our hypothesis that because the state of malnutrition and sanitation is worse in the Amazon region, there is higher prevalence of developmental delay [21,22,30]. A study in Colombia found that the two most predictive indicators for cognitive and language development in children are maternal education and quality of home environment [19]. A study in Peru that compared ASQ scores to socioeconomic indicators on early child development also found that early child development (ASQ scores) was significantly impacted by household wealth and maternal education (p<0.01 and p<0.05) [10]. The current study echoed the finding regarding the impact of maternal education (p = 0.009), but did not identify statistically significant association between wealth and child development (p = 0.07). One reason for this difference may be due to lack of diversity of wealth gradients in the rural Amazonian communities. In the communities, houses are very similar, access to education and social support are nearly the same for all people within the community, and ownership of property and use of community resources is often communal.

The primary drivers for healthy child development are good nutrition and environmental stimulation [8,10,15,21,31]. Environmental stimulation includes activities to facilitate language development, fine and gross motor skills, and problem solving. Stimulation often comes from caretaker-child interactions and households with appropriate toys, books, and other material to facilitate activity and learning [32,33]. Many of the descriptive results of the social determinants survey showed behavioral and environmental factors that can increase the risk of malnutrition. For example, 20% of mothers did not take any maternal vitamins, 24% of caretakers did not give their child micronutrient supplements, 60% have never received dewormer medication, 18% get their drinking water from the river, and 49% have had diarrhea in the last month. The significant associations found in this study further support theories of causal connection between determinants that lead to malnutrition also contributing to developmental delay [7]. We found a significant association between developmental delay and factors that affect nutrition, such as access to safe drinking water (p<0.001) and home visits by community health agents (p = 0.013). The families in the Amazon region could greatly benefit from more healthy child-rearing practices, utilization of local health services, and consumption of nutrient supplements. However, families in the region often have limited knowledge of risk factors, have limited access to health centers due to distance and cost of travel, heath centers are often under-stocked and under-staffed, and many families still hold traditional health beliefs that can undermine the value of health care visits [34–36]. Further study is needed to better understand the barriers to more healthy practices and utilization of the rural health services.

The Community Health Agent program in the communities was created to help bring health education to the doorstep of the caretakers in distant communities. Children’s healthy development depends on healthy physical development and intensive caretaker-child interaction, caring interaction with family and other adults, and preventive and basic health care [37]. The community health agent program helps create supportive environments for children by teaching caretakers about healthy practices and early child stimulation. They are increasingly being incorporated into rural health systems because they delivery health messages in a culturally relevant manner, require less training, require less pay, and are often more dedicated to the community than traditional health center technicians [38]. With the positive association of the community health agent program on child development displayed in this study, the findings suggest continuing and scaling such programs to serve rural, high-risk communities.

## Study limitations

The study design is cross-sectional and so the correlations that were identified do not have predictive validity. Another limitation is that the ASQ has not been validated in the Amazon region of Peru. There have been no child development exams validated in the region. However, test-retest evaluations in the region showed promising results, and several other large studies showed similar results as the current study when using the ASQ-3 [10,14,18]. Another limitation is the use of the cut-off scores provided by the ASQ-3 to designate developmental delay. The cut-off scores have not been validated in the Amazon region, and so the developmental behavior of the children is being compared to developmental behavior of children in more developed regions. Additional limitations from the study were created by the selection criteria for communities and individual participants. The communities were not chosen at random, but were elected by the mayor of each district. There may be essential differences between the communities that were elected and other communities. We can therefore not generalize the results to the entire population in the region. The individuals within the communities were chosen by consensus, however, not all families were present in the community during the surveys. Several families traveled away from the community for business, school, vacation, or other reason. It is not possible to know if there is an essential difference between families that stayed in the communities and those who traveled. The difficultly in estimating the size of the population in each community, and therefore the needed sample size may also present a limitation. Regardless of the limitations, this study provides a first look at descriptive data regarding child development in the region and the social determinants that are associated.

## Conclusion

The current study shows that several children are failing to reach their development goals for their age in the Amazon region of Peru (26.7%). The study also revealed that many communities have poor sanitation infrastructure and many caretaker’s do not know how to best provide a healthy and stimulating environment for their children. Mother’s education level, age of mother at child’s birth, visits by community health agents, and access to clean drinking water were the most powerful predictors for child development. By analyzing the social determinants associated with developmental delay we can prioritize community development issues to address. We can see that providing appropriate infrastructure for health and sanitation in the community and good health education are essential to good child development.

## Supporting information

S1 Annex Map of study locations. (TIF)
