# Supplementary figures and images for: Developmental delay in the Amazon: The social determinants and prevalence among rural communities in Peru
Source: PLoS One. 2017 Oct 12;12(10):e0186263. doi: 10.1371/journal.pone.0186263 (PMC5638337; doi:10.1371/journal.pone.0186263)

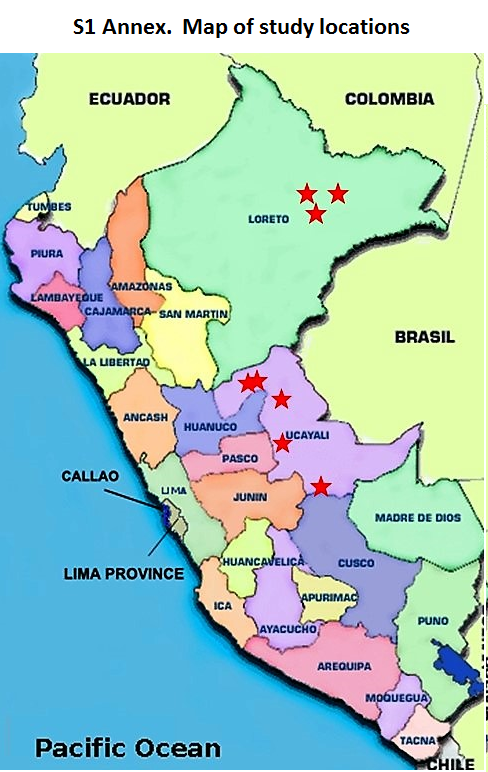

Supplement: S1 Annex — (TIF) [file pone.0186263.s001.tif]
